# An HIV epidemic is ready to emerge in the Philippines

**DOI:** 10.1186/1758-2652-13-16

**Published:** 2010-04-22

**Authors:** Anna C Farr, David P Wilson

**Affiliations:** 1 National Centre in HIV Epidemiology and Clinical Research, University of New South Wales, Sydney, Australia

## Abstract

**Background:**

The state of the HIV epidemic in the Philippines has been described as "low and slow", which is in stark contrast to many other countries in the region. A review of the conditions for HIV spread in the Philippines is necessary.

**Methods:**

We evaluated the current epidemiology, trends in behaviour and public health response in the Philippines to identify factors that could account for the current HIV epidemic, as well as to review conditions that may be of concern for facilitating an emerging epidemic.

**Results:**

The past control of HIV in the Philippines cannot be attributed to any single factor, nor is it necessarily a result of the actions of the Filipino government or other stakeholders. Likely reasons for the epidemic's slow development include: the country's geography is complicated; injecting drug use is relatively uncommon; a culture of sexual conservatism exists; sex workers tend to have few clients; anal sex is relatively uncommon; and circumcision rates are relatively high.

In contrast, there are numerous factors suggesting that HIV is increasing and ready to emerge at high rates, including: the lowest documented rates of condom use in Asia; increasing casual sexual activity; returning overseas Filipino workers from high-prevalence settings; widespread misconceptions about HIV/AIDS; and high needle-sharing rates among injecting drug users.

There was a three-fold increase in the rate of HIV diagnoses in the Philippines between 2003 and 2008, and this has continued over the past year. HIV diagnoses rates have noticeably increased among men, particularly among bisexual and homosexual men (114% and 214% respective increases over 2003-2008). The average age of diagnosis has also significantly decreased, from approximately 36 to 29 years.

**Conclusions:**

Young adults, men who have sex with men, commercial sex workers, injecting drug users, overseas Filipino workers, and the sexual partners of people in these groups are particularly vulnerable to HIV infection. There is no guarantee that a large HIV epidemic will be avoided in the near future. Indeed, an expanding HIV epidemic is likely to be only a matter of time as the components for such an epidemic are already present in the Philippines.

## Review

Southeast Asia is experiencing numerous and diverse HIV epidemics that are evolving at varying rates, in different population groups, and in different geographical areas. Approximately 5 to 10 million people are living with HIV in Asia, with prevalence estimates of well over 1% among adults in numerous countries [1]. Yet there are some settings in which HIV prevalence has remained relatively very low.

The Philippines is one of the exceptional countries that has not faced a large HIV epidemic. It is important to understand the reasons for the disparate nature of HIV in this country in order to ascertain whether lessons can be learnt for effective control in other settings and to ensure that a large HIV epidemic does not emerge in the Philippines. The first recorded case of HIV infection in the Philippines was in 1984 [2-10]. Since then, the country has maintained an HIV prevalence of less than 0.1%, even among populations at high risk [3,5,7,9], with a cumulative total number of HIV diagnoses of just over 3300 [11]. In this paper, we attempt to evaluate the current epidemiology and public health response to identify factors which could account for the "low and slow" development of the HIV epidemic in the Philippines, as well as to review behavioural and epidemiological conditions that may be of concern for facilitating an emerging epidemic.

The geography of the Philippines may be one of the first reasons for the slow spread of HIV. The Philippines is an archipelago of more than 7000 islands and islets; its complicated geography and separateness from mainland Asia could aid in shielding it from the larger regional epidemic [5,9,12,13].

Additionally, the initial core group of people usually affected with HIV in Asian epidemics is not present to a large extent in the Philippines. Most HIV epidemics in southeast Asian settings initially establish among injecting drug users (IDUs) [14]. However, there are very low numbers of IDUs in the Philippines compared with most other southeast Asian countries [5,9,13,15]. At present, there are only an estimated 10,000 IDUs in the Philippines [13] (out of its population of ~90 million people; that is, 0.01%). In comparison, neighbouring Thailand, China and Indonesia have estimated IDU populations sizes (and population proportions) of 160,000 (0.38%), 1,800,000 (0.25%) and 219,000 (0.14%), respectively [16].

There also exists a culture of relative sexual conservatism in the Philippines [9,17]. There are limited data available on sexual partner acquisition in the Philippines, and detailed behavioural sentinel surveillance data are not widely released [18]. The only reference to sexual partner rates of which we are aware is from a previous Philippines National AIDS Council Report, which indicates that the majority of the male population has only one sexual partner at any time and relatively low partnership breakup rates [19]. Although the validity of this statement should be questioned until solid data have been evaluated, this suggests that sexual conservatism exists in the Philippines relative to neighbouring countries.

The limited reporting available from behavioural surveillance conducted a number of years ago suggests that Filipinos tend to have fewer sexual partners than their counterparts in countries with higher HIV/AIDS rates [20]. For example, sex workers in the Philippines tend to have fewer clients, an average of between two and four per week compared with ~15 in many other settings [5,13,15,21,22]. Although this does not indicate levels of sexual activity in the general population, it is indicative of less sexual mixing outside regular partnerships.

However, fewer sexual partners is not necessarily a clear indicator of a smaller epidemic as reflected in China's expanding HIV epidemic despite reported sexual partner acquisition rates being similarly low [23]. One could expect different sexual behaviour across different social strata and thus an HIV epidemic sustained at low levels may not necessarily be a reflection of low average rates of partner change across a population.

There has also been the establishment of social hygiene clinics to allow for regular examination and sexually transmitted infection (STI) treatment for establishment-based female sex workers [5,15,22]. The prevalence of ulcerating STIs, which are believed to facilitate HIV transmission [24,25], is relatively low [13]. There is also a low occurrence of penile-anal sex in the Philippines [13] and a high rate of circumcision, ~93% [9,26], which is known to reduce the risk of males acquiring HIV in heterosexual intercourse [27-29].

Some countries, such as Vietnam, Indonesia and Papua New Guinea, have shown that a delayed HIV epidemic is possible [6,30]. While HIV prevalence has remained "low and slow" [5,6,31], the presence of many conditions for a large, increasing and generalized HIV epidemic are in place in the Philippines. These include: a low rate of condom use; unsafe injecting practices among IDUs; large migration rates; increasing trends in extramarital and premarital sex; a lack of education and common misconceptions about HIV/AIDS; and cultural factors that inhibit public discussion of issues of a sexual nature [10]. We will now expound these factors.

### Condom use

The Philippines has the lowest documented rates of condom use in Asia [2,32], at 20-30% among groups at highest risk of HIV (including sex workers) [4,5,8,17,21,33,34]. This is concerning since the vast majority of HIV transmission in the Philippines is through sexual contact [10,13,17,32]. A survey published in 2003 found that 63% of male respondents said that they had never used a condom [2]. Condom use among any extramarital partners is also rare [8].

There are various factors that may contribute to low condom use in the Philippines. A common perception is that condoms are only for birth control and not for protection against HIV and other STIs [8]. This perception is reinforced by the view that condoms are discouraged by the Roman Catholic Church. Government family planning programmes have policies against supplying condoms to unmarried people [4,35].

The cost of condoms is also relatively high [18]. The majority of the supply of condoms is from international aid agencies (e.g., USAID) [8,35]. Many female sex workers assert that "knowing" their client was reason enough to not use a condom [8]. Filipino women also tend to believe that the decision to use a condom is up to the man [8]. Men tend to feel the need to maintain their machismo image to the extent that they refuse to practice safe sex [36]. Culturally-sensitive but influential promotion of condoms appears to be an obvious gap in the Philippines HIV/AIDS response.

### Casual sex

There is anecdotal evidence among numerous media sources and organizational reports that casual sexual activity, particularly among the male population aged 15-25, has been increasing. A study from over a decade ago estimated that 55% of young men have engaged in premarital sex compared with 23% of young women [4]. While most premarital sex in the Philippines is with the person who becomes a future spouse, men are more likely to have at least one additional partner compared with women [2,4,8]. Most casual sexual encounters are unprotected [21,37,38].

However, all of this evidence is based on relatively old data. There is a great need for behavioural surveillance data to be collected and reported systematically and regularly in order to monitor risk activities, particularly around casual sex, associated with transmission.

### Injecting drug users

The most recent estimates of the size of the IDU population in the Philippines suggests that the number is relatively low [39]. However, serosurveillance of IDUs has only been available at one site, in Cebu City, and no data exist for other cities. It is possible that the actual number of IDUs is considerably greater than previously thought.

A 2004 report by the Philippines National AIDS Council estimated that only 48% of IDUs reported using sterile injecting equipment the last time they injected, and most IDUs reported that they regularly share injecting equipment [6]. A 2008 report published by the Joint United Nations Programme on HIV/AIDS (UNAIDS) indicated that the prevalence of sharing injecting equipment is still very high, with 29% of IDUs self-reporting use of an unsterile needle/syringe the last time they injected [39]. Sharing HIV-contaminated injecting equipment is an efficient mode of HIV transmission [40,41]. Given the experience of neighbouring countries, IDUs could be an important population group for the spread of HIV in the Philippines if the size of the IDU population increases.

### Overseas Filipino workers

There are approximately 7.5 million Filipinos working in 170 countries around the world, with more than 2000 workers departing from the country daily [32,42]. By participating in casual unprotected sex or other risky behaviour while overseas in higher prevalence settings, overseas Filipino workers (OFWs) become a substantial source of new HIV cases in the Philippines upon their return home.

Of all the HIV/AIDS cases reported in the Philippines, OFWs account for ~30-35% of all cases (this level has remained relatively steady over the past decade) [5,13,32]. Heterosexual sex is the dominant mode of transmission for OFWs, and the main occupations of OFWs who are infected with HIV are seafarers and domestic helpers. OFWs may be a bridge population for the spread of HIV and other STIs [32,43,44]. This population will undoubtedly be important in any HIV epidemic in the Philippines.

### HIV/AIDS education and social factors

Even though awareness of the disease is high [5], misconceptions of HIV/AIDS are widespread among health workers, as well as in the general population [2]. For example, a survey of 1200 males found that many respondents believed that antibiotics, prayer and keeping fit would protect against HIV/AIDS [32]. Many young people also believe that HIV/AIDS can be prevented or treated by a concoction of drinks, douching with detergents, interrupting coitus and washing the penis [5]. The Young Adult Fertility Survey found that a large proportion (60%) of young people believed that there was now a cure for HIV/AIDS and, as such, they could become more complacent [45].

Women in the Philippines are not largely empowered to protect themselves and negotiate for safe sex due to cultural, physiological and socio-economic factors. An estimated 43% of women have admitted to being forced into sex, and 15% believed that they were obligated to have sex with their partners [5].

Condom use is also low among the population of men who have sex with men (MSM) [5,6]. Unprotected penile-anal sex is a highly efficient mode of HIV transmission [46-51]. Discrimination, harassment and intolerance of homosexuality, particularly male homosexuality, have resulted in MSM becoming a "hidden" population group, even though 20% of reported HIV cases involve male-to-male transmission [5]. With intolerance still high, it is difficult to provide MSM with HIV/AIDS information, education and treatment.

### The current epidemiological state of HIV in the Philippines

**Figure 1:**
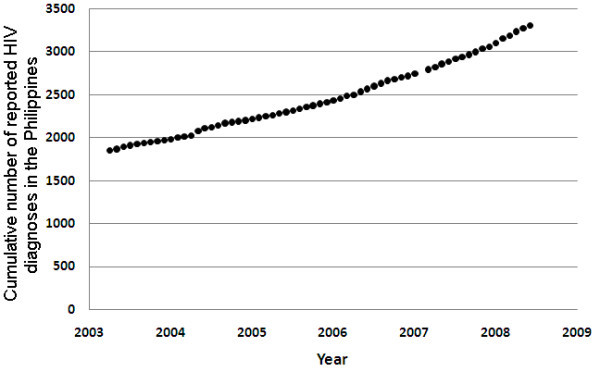
**Cumulative number of HIV diagnoses in the Philippines by month from March 2003 to June 2008**. Year on figure indicates data at the start of the year.

In this section, we present HIV/AIDS surveillance data in the Philippines and analytical findings based on monthly diagnoses reported from March 2003 to June 2008 [11]. There is a steady increase in the cumulative number of HIV notifications in the Philippines (Figure 1).

However, the trends in HIV notifications differ between the genders. The cumulative number of HIV notifications among females has been increasing at a steady rate (p < 0.0001), suggesting that incidence is approximately constant and at an endemic equilibrium. In contrast, the trend among males is not constant, incidence levels are substantially greater than in females, and the rate of new notifications is increasing (evidenced by the curvature away from linear). This suggests that there may be an emerging HIV epidemic among Filipino MSM.

**Figure 2:**
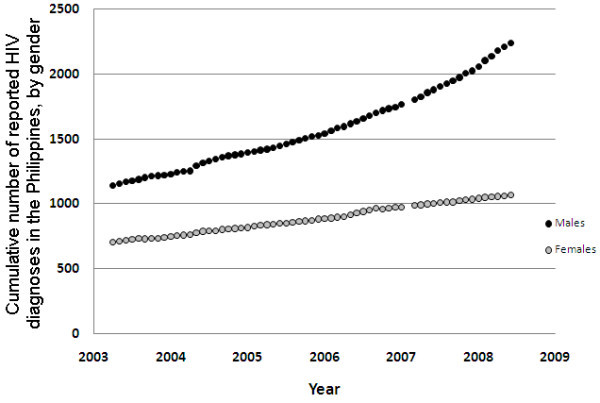
**Cumulative number of HIV diagnoses in the Philippines by month to June 2008, by gender**.

The emergence of an increasing HIV epidemic in the Philippines is evident from trends in monthly reported HIV diagnoses (Figure 2). In mid-2003, there were 10 to 15 monthly HIV notifications and there are currently 30 to 50 notifications per month; that is, a three-fold increase over five years. The trend has increased even further from 528 notifications in 2008 to 835 in 2009 (a 58.1% increase in one year) [52]. This suggests that the epidemic could be approaching a large expansion phase.

However, the divergence in HIV diagnosis rates between men and women could also reflect possible differences in testing rates. There are no data to suggest differences in testing rates, and the Philippine AIDS Prevention and Control Act of 1998 encourages HIV testing of all individuals at high risk of contracting HIV, with informed consent [53]. But this alternate explanation for the epidemic trends cannot be ruled out until reliable testing data are available.

Diagnoses of HIV in the Philippines are notified according to various categories of likely route of exposure. These include: heterosexual contact; male homosexual contact; bisexual contact; blood transfusion; injecting drug use; needle prick injury; or perinatal exposure. Bisexual contact refers to men who have had sex with both men and women. It cannot be determined whether the initial actual transmission event was male-to-male sexual contact or transmission from an infected woman. It is more likely that the transmission was via male-to-male sexual contact due to biologically higher transmission rates, but the bisexual category accurately reflects a degree of uncertainty in the route of exposure.

**Figure 3:**
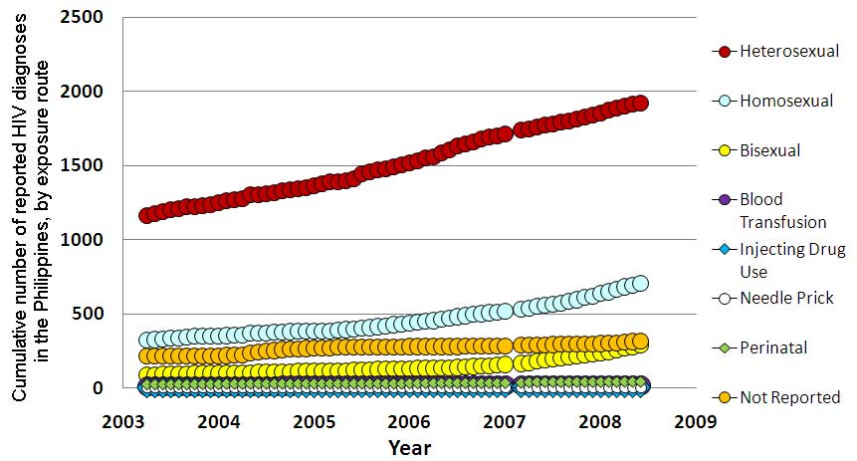
**The cumulative number of HIV diagnoses in the Philippines by month to June 2008, by route of exposure**.

The dominant mode of HIV transmission in the Philippines is sexual (~92%). But the largest increases in the rate of new HIV notifications are due to homosexual and bisexual contact, and not heterosexual contact (Figure 3). Over the period, 2003-2008, there was an increase in the monthly number of diagnoses, from 328 for homosexual contact and 92 for bisexual contact to 704 and 289, respectively; that is, respective increases of 114% and 214%. Therefore, there appears to be an increasing epidemic of HIV among men who have sex with men. The increase among bisexual men also has important consequences for the spread of HIV to the general heterosexual population. However, data on testing rates would help to elucidate the extent to which these diagnoses rates are reflective of underlying incidence.

It should be noted that some of the rise in HIV diagnoses could be attributable to an increase in testing rates. This is evident by the decreasing proportion of all HIV cases that are detected with AIDS disease: ~33% of diagnoses in 2003 were in AIDS stage disease and this has decreased to ~24%. However, the disproportionate trend in diagnoses between genders and between different routes of exposure strongly suggests that the trends in diagnoses reflect actual trends in population incidence. But since a substantial proportion of infections is detected in late-stage disease, it is likely that the majority of all HIV cases are currently undiagnosed in the Philippines [5].

**Figure 4:**
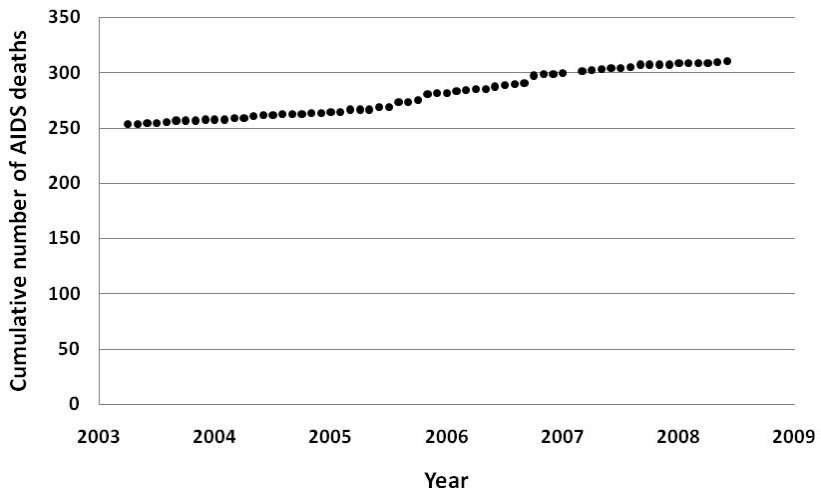
**Cumulative number of AIDS deaths by month from March 2003 to June 2008**.

The cumulative number of AIDS deaths is increasing approximately constantly (p < 0.0001), suggesting that AIDS death rates are relatively constant (Figure 4). It could be expected that there will be a delay of a number of years before the rise in HIV diagnoses translates to a rise in AIDS-related deaths.

AIDS is now a reversible HIV-related condition due to combination antiretroviral therapy (ART). The number of people receiving ART in the Philippines has been increasing since 2004, with a rate of approximately 10% of diagnosed cases receiving treatment in 2006, and ART coverage has now increased to approximately 30% [10,54]. But this is still considerably less than desirable levels. Universal treatment access for HIV-infected people is becoming a reality in some of the poorest countries of the world. Since HIV is relatively contained in the Philippines, there is the opportunity to substantially scale up treatment access before the number of HIV cases increases out of control. Treatment should be universal for HIV-positive pregnant women for preventing mother to child transmission (PMTCT) [55]. However, PMTCT is relatively uncommon in the Philippines.

One of the reasons for such low rates of ART is that funding for such care and treatment of HIV-infected persons makes up a mere 1.6% of the Philippines HIV/AIDS budget [56]. While expenditure on treatment and care is currently low, the Philippine National AIDS Council's 4^th ^AIDS Medium Term Plan and its country report for the period, January 2006 to December 2007, to the United Nations General Assembly Special Sessions (UNGASS) states that it will endeavour to improve access to treatment, care and support to HIV-infected persons [13,21]. Treatment not only sustains life among HIV-infected people, but by reducing their viral loads, it reduces infectiousness. At the population level, this would likely prevent considerable numbers of secondary transmissions of HIV [57-59].

**Figure 5:**
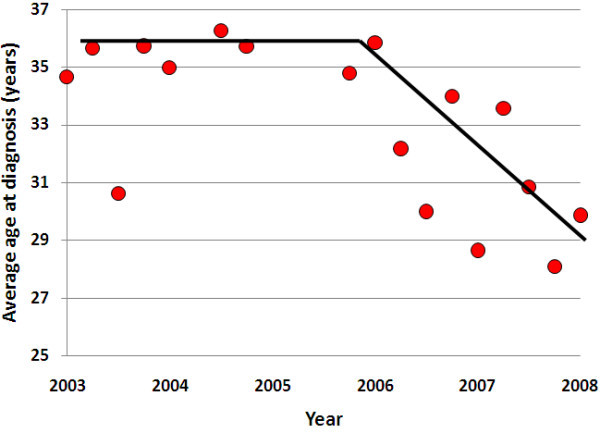
**Trend in the average age at HIV diagnosis for three-monthly notifications in the Philippines**.

The average age at HIV diagnosis in the Philippines was ~35-36 years prior to 2005, but recently, the average age at diagnosis has been decreasing (p = 0.0067) (Figure 5). It is now ~29 years of age. Although it is possible that increased testing rates mean infections are detected earlier, the extent of decrease in ages cannot be attributable to changes in testing rates.

The trend in decreased age at diagnosis is likely to reflect a decrease in age at infection. Younger age groups tend to have greater sexual activity. The fact that the average age is decreasing is a strong indicator that HIV incidence could increase substantially in the future in the Philippines. This trend is also in contrast to most other settings where epidemics are being controlled [60]. However, younger age is not necessarily indicative of greater sexual activity among all population groups, particularly among men who have sex with men, as suggested from other settings [61]. As men who have sex with men are the population group greatest affected with HIV in the Philippines, the decreasing age at diagnoses may not necessarily suggest a future increase in HIV.

## Conclusions

The Filipino government and other stakeholders have responded to the HIV/AIDS threat in the Philippines in a number of ways in order to circumvent a large HIV epidemic from arising. The Philippine National AIDS Council (PNAC) was created in 1992 to act as an advisory body to the President for the development of policy for the control of AIDS. The PNAC consists of members from the government, public, civil society, private sector and non-governmental organizations (NGOs), and is the central advisory, planning and policy-making body for the comprehensive and integrated HIV/AIDS prevention and control programme [5]. But its small budget has limited its ability to instigate implementation of large intervention and education campaigns.

The official response of the Philippines Government to the HIV threat was to enact the Philippine AIDS Prevention and Control Act of 1998 (Republic Act No. 8504) [53]. This Act was enacted by Congress after a long process of deliberation and advocacy by the PNAC and other stakeholders [19]. The Act called for: a comprehensive nationwide HIV/AIDS educational and information campaign; full protection of the human rights of known and suspected HIV-infected persons; promotion of safe and universal precautions in practices and procedures that carry risks of HIV transmission; the eradication of conditions that aggravate spread of HIV infection; and recognition of the important role that affected individuals could have in promoting information and messages about HIV/AIDS. The Act also states that local governments are to provide community-based HIV/AIDS prevention, control and care services.

While the Act is a step in the right direction, it is far from effective due to a lack of monetary commitment from the government, relying heavily on NGOs for funding for HIV/AIDS education and prevention programmes, and the current government's seemingly unwilling attitude to promote wide condom use for fear of angering the Roman Catholic Church [35]. Its statements are also broad and do not outline targeted strategies with specific goals.

Other programmes have also been established for monitoring the spread, understanding key epidemic drivers and planning the control of HIV in the Philippines. There are currently four types of surveillance systems in place in the Philippines:

1. The HIV/AIDS Registry was established in 1987 and is a passive surveillance system. It continuously records Western Blot-confirmed HIV cases reported by hospitals, laboratories, blood banks and clinics that are accredited by the Department of Health.

2. The HIV Sentinel Surveillance System (HSSS) was established in 1993 with a grant from the USAgency for International Development (USAID). It monitors 10 key cities: Baguio City, Angeles City, Iloilo City, Zamboanga City, Pasay City, Quezon City, Cebu City, Cagayan de Oro City, Davao City and General Santos City. It pays particular attention to establishment-based female sex workers, freelance female sex workers, MSM and IDUs [3,6,32].

3. Behavioral Sentinel Surveillance was added at the 10 HSSS sites in 1997 and is a systematic and repeated cross-sectional survey of behaviour related to the transmission of HIV and other STIs [3,32,62]. Its major purpose is to detect trends among vulnerable populations and groups at high risk whose behavioural change would have the greatest impact on the HIV epidemic.

4. The Sentinel STI Etiologic Surveillance System was set up in December 2001, but made operational in 2003. It monitors STI trends that could guide programme interventions to prevent the transmission of HIV.

These surveillance systems have been monitoring the progress of HIV in the Philippines and have provided valuable data to inform appropriate response measures.

The PNAC's 4^th ^AIDS Medium Term Plan for 2005 to 2010 is one of the plans that utilized data from the surveillance systems [5,21]. This plan aligns with the Philippines AIDS Prevention and Control Act, with the aims of scaling up and improving the quality of preventive interventions and the quality of treatment, care and support services for people infected with and affected by HIV/AIDS. It also aims to integrate stigma reduction measures in the preventive treatment, care and support services and in the design of management systems.

The current state of HIV in the Philippines is not attributable to any one factor. While the Philippines response is associated with effectively controlled levels of HIV, there is no guarantee that a large HIV epidemic will be avoided in the near future. Indeed, an expanding HIV epidemic is likely to be only a matter of time as the components for such an epidemic are already present in the Philippines.

Mathematical modelling studies have shown that even in countries where overall HIV prevalence has remained relatively low (e.g., Bangladesh), moderate changes in behaviour or HIV infections could initiate a large epidemic that may otherwise have taken numerous decades to develop [63,64]. Current data from the PNAC show that young adults, men who have sex with men, male and female sex workers, injecting drug users, overseas Filipino workers, and the sexual partners of people in these groups are particularly vulnerable to HIV infection [13].

The current behavioural, social and epidemiological conditions suggest that an HIV epidemic in the Philippines may be unavoidable in the near future. The number of diagnoses is increasing, particularly due to homosexual and bisexual contact; there are low condom-use rates; and the age at diagnosis is decreasing. The underlying cause of these symptoms needs to be addressed in order to prevent an emergent epidemic. The promotion of HIV prevention and education messages is underfunded and has been relatively ineffective. It is recommended that more investment be made into these programmes in order to maintain the "low and slow" development of HIV in the Philippines.

## Competing interests

The authors declare that they have no competing interests.

## Authors' contributions

ACF conducted the extensive literature search, collated available data, produced the figures and wrote the first draft of the manuscript. DPW conceived and supervised the review project and contributed to the writing of the manuscript. Both authors read and approved the final manuscript.
